# Perinatal mental health literacy: knowledge, attitudes, and help-seeking among perinatal women and the public – a systematic review

**DOI:** 10.1186/s12884-022-04865-y

**Published:** 2022-07-19

**Authors:** Daria Daehn, Sophie Rudolf, Silke Pawils, Babette Renneberg

**Affiliations:** 1 Department of Clinical Psychology and Psychotherapy, Freie Universitaet Berlin, Habelschwerdter Allee 45, 14195 Berlin, Germany; 2 Department of Medical Psychology, University Hospital Hamburg-Eppendorf, Hamburg, Germany

**Keywords:** Mental health literacy, Perinatal mental health, Stigma, Help-seeking

## Abstract

**Background:**

The perinatal period is a time of increased vulnerability to mental health problems, however, only a small proportion of women seek help. Poor mental health literacy (MHL) is a major barrier to seeking help for mental health problems. This study aimed to collect the existing evidence of MHL associated with perinatal mental health problems (PMHP) among perinatal women and the public. This review analysed which tools were used to assess perinatal MHL as well as the findings concerning individual components of perinatal MHL.

**Methods:**

Four electronic databases (PubMed, PsycINFO, Web of Science, and CINAHL) were analysed from their inception until September 1, 2020. Not only quantitative studies reporting on components of MHL (knowledge, attitudes, and help-seeking), but also studies reporting overall levels of MHL relating to PMHP were taken into account. Two independent reviewers were involved in the screening and extraction process and data were analysed descriptively.

**Results:**

Thirty-eight of the 13,676 retrieved articles satisfied the inclusion criteria. The majority of selected studies examined MHL related to PMHP in perinatal women (*N* = 28). The most frequently examined component of MHL in the selected data set was help-seeking. A lack of uniformity in assessing MHL components was found. The most common focus of these studies was postpartum depression. It was found that the ability to recognize PMHP and to identify relevant symptoms was lacking among both perinatal women and the public. Perinatal women had low intentions of seeking help for PMHP and preferred seeking help from informal sources while reporting a variety of structural and personal barriers to seeking help. Stigmatizing attitudes associated with PMHP were found among the public.

**Conclusions:**

There is a need for educational campaigns and interventions to improve perinatal MHL in perinatal women and the public as a whole.

**Supplementary Information:**

The online version contains supplementary material available at 10.1186/s12884-022-04865-y.

## Background

Pregnancy and early motherhood often signal a time of joy and excitement but also a time of massive change and challenges. During this period, women are especially vulnerable to developing perinatal mental health problems (PMHP) (i.e., mental health problems that manifest during pregnancy and up to 1 year after delivery) [1, 2]. The most prevalent mental disorders are perinatal depression and anxiety disorders. Globally, approximately 17% of women suffer from postpartum depression (PPD), which can be distinguished from temporary postpartum blues, a milder and shorter form of depressive symptoms [3, 4]. Less prevalent are bipolar disorders and postpartum psychosis with postpartum psychosis occurring in 0.1–0.2% of childbearing women [5]. All PMHP represent a public health concern due to their impact on the health of mothers and their infants. Negative associations between PMHP and behavioural and cognitive development of children up to adolescence highlight the importance of adequate and timely treatment [6, 7]. Often, however, PMHP remain undiagnosed and subsequently untreated. In the case of PPD, only 6.3% of women receive adequate treatment [8]. Unfortunately, even when perinatal health services are available, women in the perinatal period seek less help compared to women in other life periods [9, 10]. Evidence suggests that one factor influencing help-seeking rates is mental health literacy [11]. As such, poor perinatal mental health literacy might play an important role in the low healthcare utilization of perinatal women [12].

Mental health literacy (MHL) was initially defined as “[…] knowledge and beliefs about mental disorders which aid recognition, management or prevention” [13]. A more recent operationalization of the MHL concept by Kutcher et al. additionally includes the sub-components attitudes and help-seeking [14]. This definition does not only add the concept of stigma but also the concept of help-seeking efficacy to the definition of MHL. Low MHL has been identified as one of the reasons for the limited use of mental health services [14]. However, not only help-seeking efficacy but also help-seeking attitudes have been shown to be predictors of help-seeking intention and behaviour. To emphasize the concept of help-seeking attitudes as suggested by Chao et al. [15] we expanded the concept of MHL beyond the definition of Kutcher et al. to capture a wide range of help-seeking factors (e.g., intentions, barriers) for the purpose of this review.

The aim of this review was to summarize research on a broad range of perinatal MHL components in both perinatal women and the public. Inaccurate notions of mental health in perinatal women can impede early detection and treatment of their mental health problems. Therefore, perinatal MHL is an important factor influencing the recognition, diagnosis, and treatment of PMHP. For instance, Dennis & Lee [16], who summarized qualitative studies on postpartum depression help-seeking barriers, found lack of knowledge and the acceptance of myths to be important help-seeking barriers impeding mothers to recognize the emergence of depression. Moreover, help-seeking was shown to be influenced by stigma, shame, and the fear of being labelled mentally ill [16]. However, not only the view of perinatal women but also the public’s view regarding mental health problems in the perinatal period is an important factor to understand the decision-making, help-seeking, and healthcare utilization of women in the perinatal period [17]. To deliver effective MHL interventions it is important to consider the context that influences the impact of the interventions, including the public’s view on PMHP. Several studies suggest that the general population has poor knowledge about PPD [18, 19], which could potentially discourage women from seeking professional help.

According to Kutcher et al. [14], effective MHL interventions should improve the knowledge and help-seeking aspect of MHL and reduce stigma. As most studies that examined perinatal MHL in perinatal women and the public focused on individual aspects of MHL (e.g., knowledge component) [17, 20], the rationale for conducting this review was to synthesize findings on all aspects of perinatal MHL (knowledge, attitudes, and help-seeking). Identifying the components of MHL that are especially prone to impede help-seeking for PMHP could inform MHL campaigns and interventions. Therefore, it is important to conduct research on all components of MHL regarding PMHP in perinatal women and the public.

We summarized research on perinatal MHL expanding the concepts of Jorm et al. [13] and Kutcher et al. [14]. Our purpose was (1) to identify tools to measure PMHL components and (2) to summarize the existing evidence on MHL among perinatal women and the public with a focus on MHL components knowledge, attitudes, and help-seeking.

## Methods

This review followed the PRISMA reporting standards [21]. The PRISMA checklist is available in Additional file 1. The protocol is available at https://www.crd.york.ac.uk/prospero/display_record.php?ID=CRD42020208450. We used the PEO (Population, Exposure, Outcomes) framework to specify our research questions. P: perinatal women or the public. E: PMHP (e.g., postpartum depression, prenatal depression) O: MHL components: knowledge, attitudes, help-seeking, and overall levels of MHL.

### Inclusion and exclusion criteria

Published studies in German or English were eligible for inclusion. Databases were searched from their year of inception until September 1, 2020, without geographic restriction. We included all studies assessing MHL of PMHP among perinatal women and the public. We excluded studies investigating concepts (knowledge, attitudes, help-seeking) among professionals (e.g., midwives, general practitioners). Only outcomes related to maternal - not paternal - mental health in the perinatal period were included. We included quantitative studies (e.g., cross-sectional studies, prospective cohort studies). For studies other than cross-sectional studies, only baseline results were included. Qualitative studies, reviews, and meta-analyses were excluded. If qualitative studies used open-end questions and presented their results in a quantitative manner (e.g., percentages), studies were included.

### Search strategy for identification of studies

On September 1, 2020, the databases PubMed, PsycINFO, Web of Science, and CINAHL were systematically searched. We performed a Boolean search using the concepts (1) MHL, (2) perinatal period, and (3) mental illness. We used the following keywords: (“Mental health literacy” OR “Health literacy” OR literacy OR knowledge OR attitude* OR belief* OR stigma* OR “help-seek*”) AND (prenatal OR antenatal OR pregnancy OR “before birth” OR postnatal OR postpartum OR “after birth” OR peripartum OR perinatal) AND (“mental health” OR “mental illness” OR “mental disorder” OR “psychiatric disorder” OR depression OR anxiety OR “baby blues” OR psychosis OR “bipolar disorder”) to search titles, abstracts, keywords and MeSh terms (see Additional file 2). Additional studies were identified through a manual search of the bibliographic references of the included full texts.

### Study selection and critical appraisal

We imported all identified references to the literature database EndNote and removed duplicate records of the same reports. Two reviewers independently screened titles and abstracts and subsequently screened all retrieved full texts for inclusion and exclusion criteria. The methodological quality of the included studies was independently assessed by both researchers using the following tools: (1) Included Randomized controlled trials (RCTs) were assessed by the Cochrane Collaboration’s Tool for RCTs [22]; (2) Cross-sectional studies were assessed by the Newcastle-Ottawa Scale (NOS) [23]; (3) Non-randomized studies (all cohort studies, case-control studies) were assessed by the Qualitative Assessment Tool for Quantitative Studies (QATSQ) [24] (see Additional file 3). Any disagreements between the two researchers were resolved through discussion and consensus.

### Data extraction and synthesis

Two researchers extracted the data according to a developed data extraction form. To extract numerical data from plots, we used WebPlotDigitizer [25]. The extracted data included study information (e.g., authors, publication year); study characteristics (e.g., study design, sampling method); participant characteristics (e.g., sex, age) and outcomes: (1) tools to measure perinatal MHL components and (2) perinatal MHL components and levels of perinatal MHL. The extracted MHL components extended the definitions of MHL by Jorm et al. [13] and Kutcher et al. [14] and included: (a) knowledge of PMHP (recognition, symptoms, causes, first aid, intervention, and preventive measures), (b) attitudes towards PMHP (stigmatizing attitudes and beliefs), (c) help-seeking attitudes (preferred treatment, preferred source of help, barriers and facilitators) & intentions. Data were descriptively analysed.

## Results

**Fig. 1:**
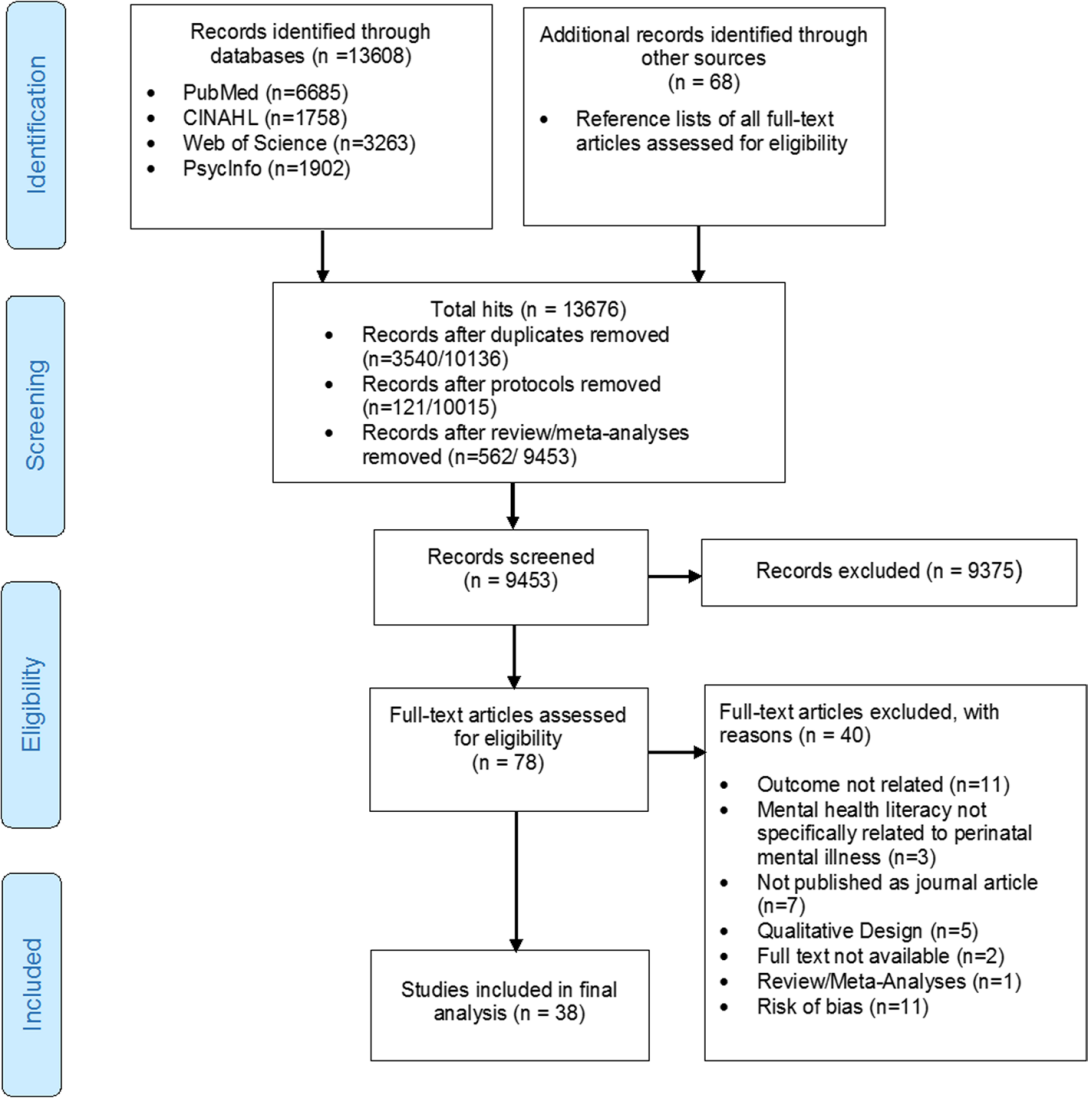
Study selection flowchart

Of the 13,608 references retrieved from the databases and the 68 references retrieved from the reference sections of included studies, we identified 78 full texts of potentially eligible articles. After full-text screening and critical appraisal, 38 eligible studies remained and were included (see Fig.1).

### Characteristics of studies

Study characteristics and components of perinatal MHL are shown in Table 1.

**Table 1:** Study characteristics and components of perinatal mental health literacy (*N =* 38)

Authors	Country (setting)	Study Design / sampling method	method of data collection (setting)	Sample size	Illness Studied	Type of participants	Sex	Mean Age (SD)	Outcomes		
									Know-ledge	Atti-tudes	Help-Seeking
Ayres 2019 [26]	Australia	Cross sectional study/convenience sampling	Questionnaire (hospital)	218	Perinatal mental health problems	Pregnant women	Female (100%)	N/A			h, k
Azale 2016 [27]	Ethiopia	Cross sectional study / community sampling	Face-to-face interview (participants’ homes)	385	Postpartum depression	Postpartum women with potential depressive disorder (PHQ-9 > =5)	Female (100%)	28.8 (5.2)	c,g		h, i, k
Barrera 2015 [28]	Latin America	Cross sectional study/ convenience sampling	Internet survey	1760	Perinatal depression	Pregnant women	Female (100%)	28.3 (5.7)			i, k
Bina 2014 [29]	Israel	Prospective longitudinal study / convenience sampling	Telephone survey	88	Postpartum depression	Postpartum women who screened positive for PPD (EPDS> = 9)	Female (100%)	29.7 (5.86)			i, k
Branquinho 2019 [30]	Portugal	Cross sectional study/ volunteer and snowball sampling	Internet survey	621	Postpartum depression	General public (Perinatal women excluded)	Female (88.1%) Male (11.9%)	32.05 (9.99)	a, b, c, g	l	
Branquinho 2020^m^ [31]	Portugal	Cross sectional study / volunteer sampling	Internet survey	621	Postpartum depression	General public (Perinatal women excluded)	Female (88.1%) Male (11.9%)	32.05 (9.99)		l	
Buist 2005 [32]	Australia	Cross sectional study / convenience sampling	Questionnaire (postnatal check-up)	420	Perinatal depression	Postpartum women	Female (100%)	N/A	a, g		i
Buist 2007 [20]	Australia	Cross sectional study/ Convenience sampling	Questionnaire (postnatal check-up)	394	Perinatal depression	Postpartum women	Female (100%)	N/A	a		
DaCosta 2018 [33]	Canada	Cross sectional study / volunteer sampling	Internet survey	652	Perinatal mental health problems	Nulliparous Pregnant women	Female (100%)	32.0 (4.3)			i, k
Dunford 2017 [34]	UK	Cross sectional study/ volunteer sampling	Internet survey	185	Postpartum depression	Postpartum women	Female (100%)	31 (5.16)		l	k
Fonseca 2015 [35]	Portugal	Cross sectional study / volunteer sampling	Internet survey	198	Perinatal depression	Perinatal women with a positive screen for depression	Female (100%)	30.59 (4.63)			h, k
Fonseca 2017 [36]	Portugal	Cross sectional study / volunteer sampling	Internet survey	231	Perinatal depression, anxiety	Perinatal women in a romantic relationship	Female (100%)	29.99 (5.07)			h
Fonseca 2018 [37]	Portugal	Cross sectional study / volunteer sampling	Internet survey	226	Perinatal depression & anxiety	Perinatal women	Female (100%)	30.08 (4.12)		l	h, k
Ford 2019 [38]	UK	Cross sectional study / volunteer sampling	Internet survey	71	Perinatal mental health problems	Postpartum women with symptoms of distress	Female (100%)	32.85 (5.69)			k
Goodman 2009 [39]	United States	Cross sectional study / convenience sampling	Questionnaire (Obstetric clinics)	509	Perinatal depression	Pregnant women in the third trimester	Female (100%)	31.6 (5.32)			I, j, k
Goodman 2013 [40]	United States	Cross sectional study / Convenience sampling	Questionnaire (Hospital)	60	Perinatal depression	Pregnant women	Female (100%)	25.49 (5.19)			h, j, k
Henshaw 2013 [41]	United States	Cross sectional study / Convenience sampling	Telephone survey	Baseline: 36; 6 week follow-up: 28	Perinatal depression & anxiety	Perinatal women	Female (100%)	28.4 (4.69)	c		h
Highet 2011 [18]	Australia	Cross sectional study/ random sampling	Telephone survey	1201	Perinatal depression	General public	Female (73.8%) Male (26.2%)	N/A	a, b, c	l	I
Holt 2017 [42]	Australia	Cluster randomised controlled trial / cluster sampling	Telephone survey	541	Postnatal depression & anxiety	Postpartum women	Female (100%)	Intervention group (IG): 31.5 (4.7); routine care (CG): 32.1 (4.6)			i, k
Kim 2010 [43]	United States	Cross sectional study / Convenience sampling	Telephone survey	51	perinatal depression	Perinatal women at risk for depression	Female (100%)	N/A			k
Kingston 2014a [44]	Canada	Cross sectional study / convenience sampling	Telephone survey	1207	Perinatal depression & anxiety	General public	Female (50%) Male (50%)	N/A			i, j
Kingston 2014b [17]	Canada	Cross sectional study / random sampling	Telephone survey	1207	Perinatal depression & anxiety	General public	Female (50%) Male (50%)	N/A	b, c		
Logsdon 2018a [45]	United States	Cross sectional study / Convenience sampling	Interview (Academic health sciences center)	50	Postpartum depression	Postpartum Latina immigrant mothers	Female (100%)	27.9 (6.2)	f		h. j, k
Logsdon 2018b [46]	United States	Pretest-posttest design /convenience sampling	Questionnaire (Community organizations; home visits)	Control group: 138; intervention group: 154	Postpartum Depression	Adolescent postpartum women	Female (100%)	Control group (CG): 18.2 Intervention group (IG): 17.9			h
Mirsalimi 2020^m^ [47]	Iran	Cross sectional study Convenience sampling	Questionnaire (hospital)	692	Postpartum Depression	Perinatal women	Female (100%)	27.63 (5.46)			i
O’Mahen 2008 [48]	United States	Cross sectional study / Convenience sampling	Telephone survey	108	Perinatal depression	Pregnant women	Female (100%)	N/A			i, j, k
O’Mahen 2009 [49]	United States	Longitudinal study/ convenience sampling	Telephone survey	82	Perinatal depression	Pregnant women (> = 10 EPDS)	Female (100%)	30.02 (4.9)	c		k
Patel 2011 [50]	United States	Cross sectional study/ volunteer sampling	Internet survey	100	Perinatal depression	Perinatal women	Female (100%)	31 (5.0)			j
Prevatt [51]	United States	Cross sectional study/ convenience and snowball sampling	Internet survey	211	Postpartum mood disorder symptoms	Postpartum women	Female (100%)	32.99 (4.10)			i, k
Ride 2016 [52]	Australia	cross-sectional discrete choice experiment/ convenience sampling	Internet survey	217	Perinatal depression & anxiety	Perinatal women	Female (100%)	32.0			h, j, k
Sealy 2009 [19]	Canada	Cross sectional study/ Community sampling	Telephone interview	8750	Postpartum depression and baby blues	General public	Female (55.8%) Male (44.2%)	N/A	b, g		i
Sleath 2005 [53]	United States	Cross sectional study / convenience sampling	(County health department)	73	Prenatal depression	Pregnant women 12–32 weeks prenatal	Female (100%)	23.6 (4.9)			j
Small 1994 [54]	Australia	Case control study	At home	Case group: 45; control group: 45	Postpartum depression	Postpartum women	Female (100%)	N/A	c		i
Smith 2019 [55]	Australia	Cross sectional study	Internet survey	1201	Perinatal depression & anxiety	General public	Female (51%) Male (49%)	N/A	a, b, c, g	l	h, I
Thorsteinsson 2014 [56]	Australia	Cross sectional study/ Convenience sample	Internet survey	500	Postpartum depression	General public	Female (85.4%) Male (14.6%)	33.73 (9.55)	a, c		i, j
Thorsteinsson 2018 [57]	Australia	Randomised controlled trial/ random sampling	Internet survey	212	Postpartum depression	General public (Parents)	Female (91.5%) Male (8.5%)	36.88 (8.71)		l	h
Wenze 2018 [58]	United States	Cross sectional study/ Volunteer sample	Internet survey	241	Perinatal mental health problems	General public (Parents of twins or higher order multiples)	Female (80.9%) Male (19.1%)	41.91 (10.79)			h, j, k
Zittel-Palamara 2008 [59]	United States	Cross sectional study/ Convenience sample	Telephone survey	45	Postpartum depression	Women who had or were currently experiencing PPD	Female (100%)	29.8 (7.23)			i, j, k

*N/A* Not available, *EPDS *Edinburgh postnatal depression scale [60]; a, Recognition of disorder; b, Symptoms; c, Causes; e, First aid/self-help; f, Prevention; g, Intervention; h, Help-seeking intention; i, preferred source of help; j, preferred treatment; k, barriers and/or facilitators to help-seeking; l, Stigmatizing attitudes and beliefs towards PMHP; ^m^Studies reporting on overall depression literacy levels; Studies of authors written in italic are based on the same sample

The majority of studies were cross-sectional studies using convenience sampling. Participants in 28 studies (73.7%) were women in the perinatal period who were either pregnant or had recently given birth. Seven of these studies only included women at risk of perinatal depression, women who had or were currently experiencing PPD, or women with symptoms of distress. Next to the tools used to measure PMHL, results are presented within the main categories of MHL: knowledge, attitudes, and help-seeking. Results on overall levels of PMHL can be found in Additional file 4.

### Tools to measure perinatal MHL components

The most commonly used tools to measure the knowledge component of perinatal MHL were vignette-based measures (23.1%), measures drawn from the Australian *Perinatal Depression Monitor* [18], and study-specific measures (e.g., semi-structured interviews, true/false questions). Attitudes and beliefs towards PMHP were most commonly examined by the *Attitudes about Postpartum Depression Questionnaire* [30] and the *Stigma subscale of the Portuguese version of the Inventory of Attitudes Toward Seeking Mental Health Services* [61]. Help-seeking attitudes (preferred treatment, preferred source of help, barriers, and facilitators) were most commonly assessed by showing participants a list of items and asking them to select all that applied. Help-seeking intentions were most commonly captured by single questions (e.g., *‘would you seek help if you had symptoms of postnatal depression or anxiety?’).* See Additional file 5 for details.

### Knowledge of PMHP

Results on the knowledge component of perinatal MHL are presented in Table 2.

**Table 2:** Knowledge of PMHP reported in studies

Knowledge component	Studies (***N*** = 13)	
	Public	Perinatal women
**Recognition**		
More than 50% of participants were able to recognize perinatal mental illness	Thorsteinsson 2014 [56] (PPD: 77.5%), Branquinho 2019 [30] (recognized the term PPD: 99.8%)	
Less than 50% of participants were able to recognize perinatal mental illness	Highet 2011^a^ [18] (PPD: 37.3%; stress: 10.7%; postnatal anxiety / panic attacks: 9.9%; prenatal depression: 3.7%), Smith 2019 [55]^a^ (PPD: 35.6%; Postnatal Anxiety / panic attacks: 12.8%; prenatal depression: 2.5%; prenatal anxiety/panic attacks: 21.0%)	Buist 2005 [32] (PPD: 32%); Buist 2007 [20] (PPD: 47.1%)
**Symptoms**		
PPD: negative thoughts about the baby (66.7%); sleeping and eating problems (81.5%); difficulties responding to respond to their partners and other children’s needs (85.3%); difficulties responding to their baby’s needs (77.1%); severe sadness and irritability (57.3%)	Branquinho 2019 [30]	
PPD: feeling sad/miserable (30.2%); Lack of bonding or worry about bonding with baby (26.2%); feelings of not coping (20.3%); Isolation (20.2%); Feeling tired (16.3%); Feeling stressed/anxious (15.3%); Loss of interest (11.3%); Sleeping problems (10.1%); Low self-esteem (9.8%); Mood changes (9.1%); Anger (8.3%); Weight (7.4%); Irritability (7.1%)	Highet 2011 [18]	
PPD: Women with PPD find it difficult to respond to their baby’s cues (68.6%); women with PPD find it more difficult to respond to the needs of their partner or other children (79.8%)	Kingston 2014b [17]	
PPD: sadness (63.2%); frustration/irritability (26.0%); sleep/appetite problems (20.6%); feelings of guilt toward the baby (19.0%); anxiety/fears (12.2%); harm to self or the baby (< 5.0%); hopelessness/helplessness (5.0%); social isolation (< 5.0%) baby blues: same symptoms as PPD (28.1%), not extending 2 weeks (29.9%)	Sealy 2009 [19]	
PPD: feeling sad/miserable (37.1%); fatigue/sleep problems (23.4%); lack of bonding with baby (19.5%); anger/irritability/aggression (17.2%); social isolation/withdrawal (13.5%); anxiety/panic attacks (12.8%); mood changes (9.3%); weight/appetite changes (8.7%); feelings of not coping (8.4%); loss of interest/pleasure (3.7%); self-esteem/confidence (3.3%) Postnatal anxiety: anxiety/panic attacks (17.1%); fatigue/sleep problems (13.2%); depression/sadness (9.8%); physical symptoms (9.4%); social isolation/withdrawal (8.1%); anger/irritability/aggression (6.9%); exaggerated/constant worrying (6.4%); inability to relax (6.4%); racing/intrusive thoughts (1.5%); obsessive behaviours (1.4%)	Smith 2019 [55]	
**Causes**		
PPD: Psychosocial causes (financial difficulty, and unsupportive partner and “thinking too much”) (60%)		Azale 2016 [27]
PPD: mainly caused by hormonal changes (28%); don’t know (31.7%), depression or anxiety during pregnancy (60.5%)	Branquinho 2019 [30]	
Perinatal depression / anxiety: inadequate social support (22.2%); physical/hormonal change with pregnancy (19.4%); stress (11.1%); Unemployment (8.3%); Lack of sleep (8.3%); Adjustment to parenting (8.3%); Genetics (5.6%); prior mental health issue (5.6%) (primary cause of the depressive symptoms)		Henshaw 2013 [41]
PPD: Biological causes (35.4%); Unprepared for transition to parenthood (30%); Lack of support (21.8%); Not coping with infant’s demands (17.8%); Stress/pressure (15.9%); Fatigue/lack of sleep (11.4%)	Highet 2011 [18]	
Prenatal depression / anxiety: history of anxiety or depression (57.2%) PPD: prior episodes of anxiety or depression in pregnancy (60.9%)	Kingston 2014b [17]	
Perinatal depression: Stress (80.5%); Hormonal changes (73.1%);state of mind (69.5%); pregnancy (65.8%); lack of sleep (46.3%); difficulty adjusting to being pregnant (43.9%); hereditary (43.9%); own behavior (39.0%); marriage or relationship problems (31.7%);other people (23.2%); having additional child (17.1%)		O‘Mahen 2009 [49]
PPD: feeling unsupported (61.7%); being isolated (61.7%); exhaustion (31.7%); physical health factors (45%); lack of time/ space for self (66.7%); material circumstances (55%); illness/death of loved one (26.7%); baby temperament (26.7%); hormones/biology (31.7%); tendency to depression (15%)		Small 1994 [54]
PPD: biological causes (34.5%); change of lifestyle (12.2%); lack of support (8.5%); not coping with parenting (9.0%); stress/pressure (7.0%); fatigue/lack of sleep (6.4%)	Smith 2019 [55]	
PPD: hormonal changes (91%); lack of sleep (88%); lack of social support (75%); day-to-day problems (54%); difficult baby (52%); genetic tendency (47%); marital problems (45%); unprepared for parenthood (45%); uninformed about parenthood (42%); financial problems (41%); low self-esteem (39%); single parent status (39%); traumatic events (37%); obstetric factors (37%); nervous person (24%); virus or infection (13%)	Thorsteinsson 2014 [56]	
**First aid / Self-help**		
Performing religious activities, discussing with significant others, thinking less about the problem, being relaxed (most frequently mentioned factors)		Azale 2016 [27]
**Prevention**		
Mental health treatment would be effective in preventing future mental health problems (58.7%)		Logsdon 2018a [45]
**Intervention**		
PPD: professional help (92.1%); psychological intervention (77.6%); help from GP (67.0%); supplements and vitamins (4.3%); support of family and friends (5.6%)	Branquinho 2019 [30]	
Prenatal depression: partner assistance (96%); Vitamins / minerals (86%); Counselling (80%); Naturopath (49%): special diet (40%); Antidepressants (22%) PPD: Counselling (93%); partner assistance (93%); Vitamins / minerals (78%); Antidepressants (54%); Naturopath (49%); Special diet (45%)		Buist 2005 [32]
PPD: Counselling (19.4%); Support group (15.6%); Antidepressants (15.5%); Talking and listening (12.1%); Psychotherapy (9.6%); Family support (7.7%); Doctor / GP; (6.6%); Don’t know (9.9%)	Highet 2011 [18]	
PPD and baby blues: Only PPD requires professional treatment (41.4%); PPD and baby blues require professional treatment (40.8%) PPD: physician/obstetrician (85.2%); Psychiatrist/mental health worker (18.4%); local health unit (11.9%)	Sealy 2009 [19]	
PPD: counselling/psychological therapy (37.7%); antidepressants (29.5%); support group; (6.5%); family support/friends (11.6%); GP/Medical professional (7.3%); help with domestic/childcare tasks (5.5%); talking and communication (3.4%); Exercise (4.0%); don’t know (26.9%)	Smith 2019 [55]	

^a^ Percentage of spontaneous responses to the question *‘what do you consider to be the major health problems which may be experienced during pregnancy /in the first year?*’ (up to 4 spontaneous responses)

#### Recognition

The ability to recognize PMHP was reported in six studies. One study conducted in an Australian community sample reported that the majority of participants were able to recognize PPD, whereas two studies among perinatal women reported that the majority of participants were unable to recognize PPD in case vignettes. Three other studies among the public assessed recognition of PMHP by the percentage of spontaneous responses to the question *‘what do you consider to be the major health problems which may be experienced during pregnancy/in the first year?*’ [18, 55] and the question *‘have you heard about PPD?’* [30]. Depression was the most commonly cited potential health problem of women in the postpartum period [18, 55].

#### Symptoms

All studies that assessed knowledge about symptoms of PMHP included participants from the public as a whole. Only a small number of participants correctly identified typical symptoms of PPD (30.2–62.2%). The percentage of women reporting difficulties in the mother-child relationship (e.g., lack of bonding, harm to the baby) as a symptom of PPD varied heavily between 5 and 77.1%. Concerning the baby blues, in one study, approximately 30% of participants correctly stated that the baby blues would not extend longer than 2 weeks [19]. In another study, symptoms of postnatal anxiety were correctly identified by less than 20%. More than 40% of those surveyed were not able to name one symptom [55].

#### Causes

Hormonal/biological changes were a frequently cited cause of PPD and perinatal depression among perinatal women and the public. Among the public, hormonal/biological changes were the most commonly cited cause of PPD. Unpreparedness for or not coping with parenthood was another frequently mentioned cause of PPD among the public [18, 55, 56]. Lack of social support was another perceived cause of PPD and perinatal depression among perinatal women and the public, with values ranging from 8.5 to 75%. However, in contrast to the public, lack of social support was the most frequently reported cause of PPD and perinatal depression among perinatal women. Other perceived causes of PPD and perinatal depression included: lack of sleep and exhaustion, depression and anxiety during pregnancy, stress, and genetic tendencies.

#### Interventions

Regarding PPD, the public most often considered professional help (e.g., counselling, psychotherapy) to be a helpful treatment. Partner/family support, on the other hand, was considered to be helpful by a small proportion of participants from the public. In contrast, in one study, a large number (93%) of perinatal women reported that partner support was helpful for PPD. Less than 30% of participants from the general public considered antidepressants to be an appropriate intervention [18, 55]. Among perinatal women, antidepressants were cited as an appropriate intervention for treating PPD by 54% of participants [32]. The same study also indicated that 78% of participants considered vitamins and minerals helpful for treating PPD. Regarding prenatal depression, partner assistance was considered helpful by almost all participants in one study (96%), followed by vitamins and minerals (86%) [32].

### Stigmatising attitudes and beliefs regarding PMHP

Results on the stigmatising attitudes and beliefs component of perinatal MHL are presented in Table 3.

**Table 3:** Stigmatizing attitudes and beliefs reported in studies

Authors	Stigmatizing attitudes and beliefs^a^	Levels of stigma^b^
Branquinho 2019 [30]	It is normal to have PPD (17.6%); women with postpartum depression cannot be good mothers (11.4%); postpartum depression is not a sign of weakness (disagreement 11.6%); women know, by nature, how to look after a baby (23.8%); women have postpartum depression because they have unrealistic expectations about caring for a baby (12.1%)	
Branquinho 2020 [31]		Attitudes towards PPD: M = 2.52; SD = 0.51^c^; Indifference to stigma: M = 0.76; SD = 0.73^d^
Dunford 2017 [34]		Indifference to stigma: M = 21.11; SD = 7.53^e^
Fonseca 2018 [37]		Indifference to stigma: M = 3.29; SD = 0.75^d^
Highet 2011 [18]	It is normal to be depressed during pregnancy (agree / strongly agree: 52%); it is normal to have PPD (agree / strongly agree: 24%); knowing how to look after a baby comes naturally to women (agree / strongly agree: 19%)	
Smith 2019 [55]	It is normal to be depressed during pregnancy (agree / strongly agree: 32%); postnatal depression is a normal part of having a baby (agree / strongly agree: 18.5%); knowing how to look after a baby comes naturally to women (agree / strongly agree: 21.6%)	
Thorsteinsson 2018 [57]		Pre-intervention personal stigma (averaged across groups): M = 6.69^f^; Pre-intervention perceived stigma (averaged across groups); M = 17.14^f^

^a^ Reported by more than 10% of participants

^b^ Studies reporting mean values without any associated standard values

^c^ Attitudes about Postpartum Depression Questionnaire (APPD-Q [30]); higher scores indicate more negative attitudes

^d^ Stigma subscale of the Portuguese version of the Inventory of Attitudes Toward Seeking Mental Health Services (IATSMHS [61]); higher scores indicate more stigma towards PPD; range 0–4

^e^ The Inventory of Attitudes Towards Seeking Mental Health Services (IASMHS [62]); stigma subscale (indifference to stigma, range 0–32)

^f^ Depression Stigma Scale (DSS [63]); 18-items; personal stigma subscale; 5-point likert scale; scale scores ranging from 0 to 72; higher scores indicate greater stigma

The most commonly reported aspects of negative or trivializing beliefs reported among the public were: ‘*it is normal to have PPD*’ and that ‘*women know by nature how to look after a baby’*. Two studies indicated that participants most often agreed with the attitude ‘*it is normal to be depressed during pregnancy’* [18, 55]. Similarly, half of an Australian community sample viewed being depressed during pregnancy as *‘a normal part of having a baby*’ [18]. In a third study, 11.4% of the participants agreed with the statement *‘women with postpartum depression cannot be good mothers*’ and 12.1% agreed with *‘women have postpartum depression because they have unrealistic expectations about caring for a baby’*. Furthermore, 11.6% of the participants disagreed with the statement *‘postpartum depression is not a sign of weakness’* [30].

### Help-seeking for PMHP

The large majority of studies (*N* = 34) reported at least one aspect of help-seeking for PMHP. Results are presented in Tables 4 and 5.

**Table 4:** Help-seeking: intentions, preferred sources and treatment

Authors	Outcomes		
	Intention to seek help	Attitudes^**a**^ Preferred /recommended source of help	Preferred treatment
Ayres 2019 [26]	36.2%		
Azale 2016 [27]	Perceived need for treatment:71.6%	*Informal: husband (61.3%); Formal: general health professional (any) (12.7%)	Modern medicine (49.8%)
Barrera 2015 [28]		* Informal: partners (82.5%); family members (75.5%); Formal: health providers (49.4%)	
Bina 2014 [29]		*Professional help users (24%): mental health professional (71%) Informal help users (62.5%): family and friends (approx. 50%)	
Buist 2005 [32]		*Informal: family (50%); Formal: GP (29.2%)	
Branquinho 2020 [31]	Help-seeking propensity: M = 3.19; SD = 0.61^b^		
DaCosta 2018 [33]		*All women: family doctor/general practitioner (9.7%) Women EPDS> = 10: family doctor/general practitioner (19.2%)	
Dunford 2017 [34]	Help-seeking propensity: M = 21.46; SD = 6.29^b^		
Fonseca 2015 [35]	Willingness to seek professional help for psychological problems: 38.4%		
Fonseca 2017 [36]	Intention to seek professional help: M = 4.48; SD = 1.60^d^		
Fonseca 2018 [37]	Intention to seek professional help: M = 4.48; SD = 1.59^d^		
Goodman 2009 [39]		Obstetric practitioner or mental health practitioner at obstetrics clinic (69.4%)	Individual psychotherapy (72.5%)
Goodman 2013 [40]	Interested in professional mental health services: 78.3%		PPD prevention: mindfulness approach (MBCT) (47.46%)
Henshaw 2013 [41]		Informal: friend or family member (83.3%); Formal: counsellor/psychologist (58.3%)	
Highet 2011 [18]		Full sample: Informal: friends and family (32%); Formal: doctor (52%); Family / friends (male: 21,1; female: 43,1); GP (male: 32%; female: 21%)	
Holt 2017 [42]		*GP (69.6%); psychologist/counsellor (52.2%)^f^	
Kingston 2014a [44]		Informal: partner (17.7%); Formal: family doctor (38.9%)	Talking to doctor or midwife (81.6%); counselling (79.8%); peer support (73.2%); parenting help (70.3%); diet/ nutritional supplements (63.2%); phone support (52.9%)
Logsdon 2018a [45]	M = 3.8; SD = 1.2^e^		First inclination: psychological treatment (73.9%)
Logsdon 2018b [46]	Baseline CG:11.5%; Baseline IG:11.9%		
Mirsalimi 2020 [47]		Informal: friends / family members (27.2%); Formal: psychologist (42.1%)	
O’Mahen 2008 [48]		Mental health specialist (85.1%); primary care physician (68.8%); obstetrician (62.5%); pastor (60.5%)	Family/friend support (89.6%); therapy (76.4%); antidepressant; (68.7%); case management (62.5%)
Patel 2011 [50]			Combination of medication and counselling (55%)
Prevatt 2018 [51]		OB-Gyn (53.4%)	
Ride 2016 [52]	77%		Pregnant women: individual counselling; Breastfeading women: Meditation; Yoga or Exersice; Non-breastfeeding women: combinded counselling and Medication. Individual counselling was consistently the highest ranked guideline-recommended treatment.^g^
Sleath 2005 [53]			Wait and get over it naturally (83.6%); counseling from a mental health professional (57.6%)^h^
Small 1994 [54]		* Informal: friends (70%); partner (66.7%); Formal: GP (65%), maternal and child health nurse (55%)	
Smith 2019 [55]	Women who would not seek help for PPD: 3.8%	Informal: family/friends (male: 19%; female: 53%); Formal: doctor (male: 43.3%; female: 50.7%)	
Thorsteinsson 2014 [56]		Informal: family (70%); friends (68%); Formal: GP (96%); counsellor (86%); community health nurse (75%); telephone counselling service (71%); social worker (60%); internet (54%); psychiatrist (53%)	Family support (88%); support group (85%); counselling/psychotherapy; (81%); relaxation/time to self (76%); sleep (74%), exercise (74%); antidepressant medication (56%); improved diet (51%)
Thorsteinsson 2018 [57]	Help-seeking propensity (averaged across groups): M = 2.92; SD = 1.73^c^		
Wenze 2018 [58]	47.8% interested in mental health treatment in the perinatal period (for stress: 32.1%; for depression: 18.8%; for anxiety: 21.9%)		Preference Ranking: 1. Individual therapy (47.9%)
Zittel-Palamara 2008 [59]		OB/Gyn (73.3%); psychiatrist (73.3%); psychologist (71.1%); primary care physician (71.1%); social workers (66.7%); paediatricians (60%); midwives (57.8%); spiritual assistance (64.4%)	Individual counselling (84.4%); medication (73.3%); In-person support group (73.3%); hospital inpatient (68.9%); online support group (66.7%)

When only mean values of help-seeking intention / propensity without any associated standard values were presented, no conclusions were drawn; * Sources of help used by help-seeking women in the study; ^a^ Reported by more than 50% of participants; if all percentages were < 50%, the highest percentage per category was reported; ^b^The Help-seeking Propensity subscale of the Portuguese version of the Inventory of Attitudes Toward Seeking Mental Health Services (IATSMHS [61]), 8 items, 4 point likert scale, higher scores higher help-seeking propensity; ^c^Inventory of Attitudes Towards Seeking Mental health Services (IASMHS [62]), 24 items, 5-point Likert scale, Subscale Scores 0–32, higher scores indicate more positive attitudes towards help-seeking; ^d^ General Help-Seeking Questionnaire (GHSQ [64]), 7 point likert scale (range 1–7); ^e^Mental Health Intention Scale (1 item [65]), scores range from 0 to 9 with higher scores representing more intention; ^g^ discrete choice experiment; ^h^treatment preferences were measured by asking women to rate how acceptable (definitely acceptable, probably acceptable, probably not acceptable, and definitely not acceptable) certain treatments would be if they felt sad

**Table 5:** Help-seeking: barriers and facilitators

Authors	Structural Barriers*	Individual barriers (Knowledge/Attitude)*	Facilitators	
Ayres 2019 [26]	Lack of time; no one to look after child while attending appointment		Encouragement by family Encouraged by midwife / GP / obstetrician	
Azale 2016 [27]	Fear of cost (56.0%); distance (50.4%)	Problem would get better by itself (76.1%); wanting to solve the problem by herself (66.7%)	Strong social support; perceived physical cause; perceived higher severity; perceived need for treatment; PHQ score; disability ^a^	
Barrera 2015 [28]		Non-help seekers: I figured that it would pass (83.8%); I didn’t think others would understand; (77.0%); I didn’t think anyone could help me (67.4%), I didn’t know what I was feeling (65.0%), I didn’t think it was that important (59.4%), I was afraid of my feelings (53.5%); I was ashamed of my feelings (50.2%); I was embarrassed of my feelings (49.8%)	current major depressive episode; income ^a^	
Bina 2014 [29]			High confidence in mental health professional, higher levels of depressive symptoms^a^	
DaCosta 2018 [33]	Being too busy (26.1%); waiting time too long (18%); cost (22.6%); not available at time required (10.4%)	Not having gotten around to it (46.1%); deciding not to seek care (24.3%); not knowing where to go (19.1%); felt help would be inadequate (16.5%)	Less severe depressive symptoms; prior consultation for mental health^a^	
Dunford 2017 [34]		Shame proneness significantly predicted negative attitudes towards help-seeking^b^		
Fonseca 2015 [35]	Not be able to afford treatment (63.7%); do not have time to go to psychology and/or psychiatry appointments (51.9%); have sanctions for missing work to go to psychology and /or psychiatric appointments; (38.6%); do not have means to travel to psychology and/or psychiatry appointments (19.3%).	Attitudinal barriers: thinking that no one will be able to help me deal with my problems (47.4%); being afraid of what my family and/or friends might think of me (32.2%); being ashamed to talk to with health professional (36.8%); being afraid that other people discover I attend psychology and / or psychiatric appointments (33.3%) Knowledge barriers: do not know if my problems are a reason to ask for help (76%); do not know what the best treatment options is (96.2%), do not know where to seek treatment (39.2%)	Higher age; single/divorced; history of psychiatric problems and treatment^a^	
Fonseca 2018 [37]		For women with significant psychological symptoms: women’s more insecure attachment representations (anxiety and avoidance) were associated with lower intentions to seek professional help^f^		
Ford 2019 [38]	Logistics of attending appointment; logistics of getting an appointment ^c^	Fear of stigma; willingness to seek help ^c^	Interpersonal relationship with healthcare professionals (healthcare professionals being empathetic and non-judgemental, having my voice heard in discussions and decisions about treatment, opportunity to build trust and respect with healthcare professionals); support from friends and family (partners who encourage women to seek help)^d^	
Goodman 2009 [39]	Cost (22.6%); no time (64.7%), no childcare (33.2%); if there were a charge, I might not be able to afford it (18.8%)	Stigma (42.5%); would not know where to find such services (26.2%)		
Goodman 2013 [40]	Cost^e^	Belief that prayer would be sufficient to help prevent depression^e^	Severity of illness (33%), pragmatics (e.g., cost, location), (29%); knowledge; social support (19%), professional encouragement (7%)	
Holt 2017 [42]		I thought I would be able to manage on my own (11.1%); I felt I should be able to manage on my own (11.1%); I did not think I needed help; (6.7%); I did not want people to know I wasn’t coping (6.1%)	antenatal anxiety, previous history of depression; self-esteem^a^	
Kim 2010 [43]	Patient level: Lack of time (25%); Used other support (25%); spontaneous improvement of symptoms (13%) Provider level: provider unavailability (56%); unresponsive provider (25%) Patient / provider interaction: Poor match to patient need (31%); patient provider fit (31%); phone tag (31%), System level: Cost/insurance mismatch (56%); geographic mismatch (19%)		Patient level: recognition of one’s own need for treatment (14%) Provider level: treatment availability (21%) System level: Cost/insurance mismatch (21%) Additional factors: referrals tailored to patient needs; (29%); specific encouragement to engage in treatment; (21%); geographic match (21%), active facilitation of the referral process (14%)	
Logsdon 2018a [45]		Attitudes towards help-seeking: seeking psychological help carries a social stigma (34.8%); people will see them in a less favourable way if they were receiving mental health treatment (23.9%); people who seek psychological treatment are generally liked less by others (34.8%); people should work out their own problems with psychological counselling as the last resort (30.4%)	More positive attitudes towards seeking professional psychological help, less social support; less perceived control^a^	
O’Mahen 2008 [48]	1.Structural Barriers (1. insurance; 2. inability to pay; 3. transportation; 4. inadequate childcare)^g^	2. Knowledge (1. not sure who to contact; 2. Do not know what treatment might be best for me) 3.Attitudes (1. lack of expressed motivation; 2. hopelessness about treatment working)^g^		
O’Mahen 2009 [49]			Belief that symptoms would last a long time^a^	
Prevatt 2018 [59]^l^	Time constraints (18%)	Stigma (19%); lack of motivation (16%)	Social support, stress^a^	
Ride 2016 [52]	Cost^m^		High social support; high levels of education; childcare; higher efficacy, past experience of treatment^m^	
Wenze 2018 [58]	Lack of time (16.6%)			
Zittel-Palamara 2008 [59]	Tried to find assistance but was unable to find resources (15.6%); PPD symptoms made it difficult to take action (13.3%), comments from health care professional that ‘this is normal’ (13.3%)	Not being sure who to speak to (15.6%), lack of PPD education (13.3%); pressure from family and friends (e.g., ‘it is normal, you are fine’) (13.3%)		

* all reported factors were mentioned by more than 10% of participants ^a^ regression analysis ^b^ The Event-Related Shame and Guilt Scales (ERSGS [66]) ^c^ above the average score of all barriers ^d^ Four factors with highest mean scores ^e^ Rated as at least “somewhat true”; Nine questions that might interfere with seeking help; Participants rated their responses on a 7-point Likert Scale from 1 (not at all true) to 7 (extremely true) ^f^ path analysis ^g^ Women’s mean rankings of barriers of greatest concern ^l^ Facilitators and Barriers to Disclosure of Postpartum Mood Disorder Symptoms to a Healthcare Provider ^m^ discrete choice experiment, mixed logit model

Although in some studies, a high proportion of women reported a need for treatment or were interested in professional health services during the perinatal period, the percentage of women who intended to seek help for PMHP was generally below 40%. However, in one study approximately three-quarters of women stated that they would seek professional help if they experienced symptoms of perinatal depression and anxiety [52].

#### Preferred source of help

Whereas perinatal women preferred informal sources of help such as family or friends in most studies, the public commonly preferred formal sources of help such as GPs. Although women preferred informal sources of help from family and friends, men would rather recommend formal sources of help [18, 55]. The most commonly preferred formal source of help was medical health professionals (e.g., GPs), followed by mental health professionals. In one study, gynaecologists and psychiatrists were both equally preferred [59]. The remaining studies did not clearly differentiate between medical professionals and mental health professionals [28, 39].

#### Preferred treatment

The most frequently reported preferred treatment type among perinatal women and the public was counselling/therapy. Treatment preferences differed between pregnant, breastfeeding, and non-breastfeeding women [52]. Pregnant women preferred individual counselling, breastfeeding women meditation, yoga or exercise and non-breastfeeding women preferred combined counselling and medication. In one study, the most commonly preferred treatment type (83.6%) was ‘*Wait and get over it naturally’* [53].

### Help-seeking barriers and facilitators

Twenty studies assessed barriers and/or facilitators to help-seeking for PMHP in perinatal women and one among parents [58].

#### Barriers

Structural, attitudinal, and knowledge-related barriers were reported (see Table 5). Among structural barriers, two main categories emerged. (1) cost of treatment and (2) inability to attend appointments due to: time constraints, logistics/transportation, childcare, distance/geographic mismatch, and unavailability of providers/resources. The most commonly reported attitudinal barriers were associated with stigma and shame. Approximately 50% of women reported that fear, shame, and embarrassment of their feelings prevented them from seeking help [28]. Moreover, shame proneness predicted negative attitudes towards help-seeking [34]. The anticipated opinion of other people (e.g., *‘I didn’t think others would understand’* or *‘being afraid of what my family and/or friends might think of me’)* and the attitude towards help-seeking (*‘wanting to manage symptoms on their own’)* were other barriers frequently mentioned by women. The knowledge barriers most frequently mentioned were not knowing where to seek help/who to contact and not knowing what the best treatment option might be.

#### Facilitators

The majority of studies assessed facilitators that predicted help-seeking intentions or behaviour. Social support was the facilitator most commonly reported. Six studies determined high support and encouragement by family/partners as a facilitator to help-seeking or symptom disclosure; however, one study found that less social support increased treatment uptake [52]. Severity of illness was another frequently mentioned facilitator. Although higher symptom severity facilitated help-seeking in most studies, one study found that women with more severe depressive symptoms reported more barriers to help-seeking [33]. Five studies found that the relationship to and confidence in mental health professionals facilitated help-seeking. For instance, in three studies encouragement by healthcare professionals was found to be a help-seeking facilitator. Past experiences of mental illness or treatment was another commonly expressed facilitator to help-seeking in five studies. For instance, women sought professional assistance more frequently if they had a history of mental health problems and treatment [35]. The attitude towards diagnosis and treatment was another facilitator. For instance, the perceived need for treatment was found to be a help-seeking facilitator [27, 43]. Moreover, two other studies found that *‘the belief that symptoms would last a long time’* predicted help-seeking behaviour [49] and that more positive attitudes towards seeking professional psychological help increased intentions to seek help [45].

## Discussion

The purpose of this study was (1) to identify tools used to measure perinatal MHL components and (2) to summarize the existing evidence on perinatal MHL with a special focus on knowledge, attitudes, and help-seeking. This review identified several aspects of perinatal MHL, which should be targeted in interventions and campaigns.

A large heterogeneity of assessment of MHL components and sub-components was found; therefore, making it difficult to compare results. For instance, some studies reported percentages of correct responses, whereas others reported the endorsement of participants with specific statements. Several studies did not provide evidence for the psychometric validity of measures or developed their own study measures. Recognition of symptoms, for instance, was assessed in several different ways, with only half of the studies using case vignettes, which is in line with the operationalization of recognition of mental disorder as the ability to identify and name a mental disorder based on a written case vignette [67]. Our results are in accordance with the research of Singh et al. who found a lack of uniformity in assessing MHL components among adolescents [68]. In the case of symptom recognition, future research should use a standardized set of vignettes. Likewise, instead of using study-specific lists of statements to assess treatment barriers for PMHP, standardized measures such as the *Perceived Barriers to Psychological Treatment (PBPT)* scale could be adapted and used in future research [69]. Regarding levels of perinatal MHL, a tool to measure postpartum depression literacy (*The postpartum depression literacy scale, PoDLiS)* within the mental health literacy framework has been developed recently [47]. Future research should employ valid and reliable measures to assess all components of perinatal MHL literacy.

Less heterogeneity was found with regard to the specific PMHP studied. Almost all studies focused on perinatal MHL in relation to perinatal depression or PPD specifically. However, incidences of other PMHP such as perinatal anxiety are high and merit clinical attention similar to that given to perinatal depression [70]. Future research assessing MHL in the context of other PMHP is warranted.

Findings on the knowledge component of perinatal MHL suggest that women and the public have a partly fragmented and differing understanding of PMHP. Although misconceptions relating to symptoms, causes, and treatment options for PMHP were found in both, perinatal women and the public, a few differences were observed. Perinatal women most commonly considered lack of social support as a cause for PMHP; however, the public most commonly attributed postnatal depression to biological factors. Importantly, biological factors are not among the most important risk factors as identified by research: antenatal depression and anxiety, major life events, lack of (partner) support, and depression history [71–74]. This misconception and possible confusion of PPD with the baby blues may explain why some stereotypes such as ‘*it is normal to have PPD’ exist* among the public. Public educational campaigns highlighting the significance of PMHP could counteract misconceptions and trivializing notions. This seems especially important considering that higher public knowledge of PMHP is associated with higher intention to recommend help-seeking [31] and might therefore influence help-seeking behaviour of perinatal women. Perinatal women most commonly reported social support as a helpful intervention and preferred informal sources of treatment. This is disconcerting because PMHP often require professional treatment [75]. Therefore, it is important to educate women that –although social factors are among the causes of PMHP– informal sources of help (such as support from the partner) may not be sufficient to effectively treat PMHP. It is important to highlight the importance of professional help and to reduce the barriers associated with formal help-seeking.

Consistent with previous research, stigma and shame were the most prevalent barriers to help-seeking in perinatal women [16]. By discussing PMHP with perinatal women, providers (e.g., gynaecologists, midwives, and obstetricians) could improve knowledge and reduce stigma and shame. Innovative treatment options such as internet-based interventions could be used to circumvent both structural and stigma-related barriers. For instance, internet-based interventions including information and cognitive behavioural strategies were shown to influence levels of depression stigma and attitudes towards PPD [63, 76, 77].

Our finding that social facilitators (such as social support and encouragement, relationships with providers, and attitudes towards mental illness) are the most commonly reported reasons to seek help has also been reported elsewhere [78]. To strengthen social support, interventions should be developed that provide strategies for reinforcing and mobilizing women’s social networks in the perinatal period; e.g. by developing a post-birth support plan [51]. This seems particularly important as the social network often tends to recommend formal rather than informal treatment and therefore may serve as an important gateway for the transition from informal to formal treatment.

### Practical implications

There is a need for campaigns and interventions to raise perinatal MHL among both, perinatal women and the public.

First, perinatal women and the public should be educated about the symptoms, risk factors, and treatment options of PMHP to increase problem recognition and service selection. Common misconceptions – such as the high attribution of PPD to biological factors and the underestimation of psychosocial causes – should be addressed. Given the important role of partners in encouraging women to seek help, it seems essential that the social network of women can recognize PMHP and understands the important role of support and encouragement as a facilitator to perinatal help-seeking. Consistent with the recommendations of Poreddi et al. [79], this review highlights the importance of educational campaigns, which aim to improve perinatal MHL by addressing prejudices and negative stereotypes associated with PPD [79]. Importantly, campaigns and interventions should not solely focus on PPD, but also raise awareness about less understood PMHP such as prenatal depression and perinatal anxiety.

Additionally, perinatal women should receive information on relevant providers and treatment options to decrease knowledge barriers to help-seeking and subsequently facilitate service selection. Ideally, healthcare providers who work directly with pregnant women and new parents (such as midwives, gynaecologists, paediatricians, and GPs) should discuss PMHP, screen for PMHP, discuss treatment options, and refer patients for treatment. However, medical professionals often lack resources or knowledge to address PMHP [80]. In addition to raising the awareness of health care professionals with the goal of increasing provider MHL and thus screening rates [81], more comprehensive approaches are needed. Given that the smartphone is the most commonly used device with internet access among perinatal women [82], developing and evaluating evidence-based content for smartphone use could be one approach to improve perinatal MHL among women and the public. Such an approach is currently evaluated (www.smart-moms.de).

Second, campaigns and interventions should focus on stigmatizing attitudes. Stigma and shame are not only a substantial barrier to help-seeking for PMHP [16], but also influence the public’s intention of recommending professional help for PMHP [31]. Given that social support and partner encouragement are important help-seeking facilitators, campaigns and interventions addressing stigmatizing attitudes towards PMHP among the public have the potential to increase the essential support from the social network and subsequently increase help-seeking rates among perinatal women.

### Strengths and limitations

To our knowledge, this study was the first systematic review to summarize findings on perinatal MHL. Moreover, this review incorporated several aspects of perinatal MHL and expanded the concept by Kutcher et al. [14] to capture a wide range of help-seeking factors (e.g., intentions, barriers). The quality of studies was appraised by using different tools recommended for use in systematic reviews. A limitation that should be mentioned is that we limited our search to studies in English and German and did not include any source of Grey literature. Therefore, this review might be subject to publication bias. Moreover, due to the substantial number of outcomes related to perinatal MHL and the heterogeneity of tools used in the studies, findings should be interpreted with caution. It should be noted that most of the included studies were conducted in Western countries. Since the experience of shame and stigma is often culturally or socially determined [83, 84], our results may not be generalizable to non-Western cultures. As PMHP also affect men [85], future research on MHL in relation to paternal PMHP, and any interactions or associations between maternal and paternal PMHP, is warranted. Additionally, future reviews with a focus on qualitative studies would be highly valuable to shed more light on the individual experiences of perinatal women and the public as a whole.

## Conclusions

In summary, a multidisciplinary approach that supports perinatal health care professionals in their role as *gatekeepers* to perinatal mental help treatment and also increases the accessibility of sensitive information about PMHP for perinatal women and the public is needed. Future research should investigate the effects of perinatal MHL campaigns and interventions on actual help-seeking behaviour.

## Supplementary Information

**Additional file 1.** Prisma 2020 Checklist.

**Additional file 2.** Search Terms. This file provides an overview of the search strategy.

**Additional file 3.** Quality Assessment. This file presents the critical appraisal of the included studies.

**Additional file 4.** Supplemental Results. This file presents the results relating to overall levels of perinatal mental health literacy.

**Additional file 5.** Tools to measure perinatal mental health literacy components. This file summarizes the tools used in the included studies [86–90].

## Data Availability

All data generated or analysed during this study are included in this published article [and its supplementary information files].

## Abbreviations

MHL: Mental health literacy
PMHP: perinatal mental health problems
PPD: postpartum depression
PRISMA: Preferred Reporting Items for Systematic Reviews and Meta-Analyses
