# Supplementary material for: Perinatal mental health literacy: knowledge, attitudes, and help-seeking among perinatal women and the public – a systematic review
Source: BMC Pregnancy Childbirth. 2022 Jul 19;22:574. doi: 10.1186/s12884-022-04865-y (PMC9295513; doi:10.1186/s12884-022-04865-y)
Supplement: Supplementary file 2 — Additional file 2. Search Terms. This file provides an overview of the search strategy. [file 12884_2022_4865_MOESM2_ESM.pdf]

Perinatal mental health literacy: Knowledge, attitudes, and help-seeking among perinatal women and the public– a systematic review

Search Terms

1. WEB OF SCIENCE SEARCH TERMS

No.	Term	Hits (date: 01.09.2020)	Notes
CONCEPT 1			
#1	"Mental health literacy" OR "Health literacy" OR literacy OR knowledge OR attitude* OR belief* OR stigma* OR "help-seek*" [Topic]	2,065,570	CONCEPT 1
CONCEPT 2			
#2	prenatal OR antenatal OR pregnancy OR "before birth" OR postnatal OR postpartum OR "after birth" OR peripartum OR perinatal [Topic]	694,427	CONCEPT 2
CONCEPT 3			
#3	"mental health" OR "mental illness" OR "mental disorder" OR "psychiatric disorder" OR depression OR anxiety OR "baby blues" OR psychosis OR "bipolar disorder" [Topic]	916,327	CONCEPT 3
#4	#1 AND #2 AND #3 (No Filter)	3,346	
#5	#1 AND #2 AND #3 (with filter)<br>• English and German	3,185	

2. PUB MED SEARCH TERMS

No.	Term	Hits (date: 01.09.2020)	
CONCEPT 1			
#1	"Health Literacy" [Mesh]	5,975	
#2	"Health Knowledge, Attitudes, Practice" [Mesh]	111,940	
#3	"Health education" [Mesh]	244,278	
#4	"Attitude to health" [Mesh]	418,910	
#5	"Help-Seeking Behavior" [Mesh]	777	
#6	#1 OR #2 OR #3 OR #4 OR #5	607,837	
#7	"Mental health literacy" OR "Health literacy" OR literacy OR knowledge OR attitude* OR belief* OR stigma* OR "help-seek*" [TIAB]	920,772	
#8	#6 OR #7	1,398,104	Concept 1

CONCEPT 2			
#9	"Postpartum Period" [Mesh]	65,200	
#10	"Pregnancy" [Mesh]	895,913	
#11	9# OR #10	918,403	
#12	prenatal OR antenatal OR pregnancy OR "before birth" OR postnatal OR postpartum OR "after birth" OR peripartum OR perinatal [TIAB]	673,243	
#13	#11 OR # 12	1,157,333	Concept 2
CONCEPT 3			
#14	"Depression"[Mesh]	119,760	
#15	"Anxiety"[Mesh]	85,659	
#16	"Mental Disorders" [Mesh]	1,243,983	
#17	"Mental Health" [Mesh]	38,814	
#18	#14 OR #15 OR #16 OR #17	1,379,500	
#19	"mental health" OR "mental illness" OR "mental disorder" OR "psychiatric disorder" OR depression OR anxiety OR "baby blues" OR psychosis OR "bipolar disorder" [TIAB]	636,389	
#20	#18 OR #19	1,646,556	Concept 3
#21	#8 AND #13 AND #20<br>(Without Filter)	7,079	
#23	#8 AND #13 AND #20<br>(With filter)<br>• English and German	6,685	

3. CINAHL SEARCH TERMS

No.	Term	Hits (date: 01.09.2020)	
CONCEPT 1			
#1	MH "Health Literacy"	4,198	
#2	MH "Health Knowledge"	30,437	
#3	MH "Health Beliefs"	13,581	
#4	MH "Health education"	26,299	
#5	MH "Attitude to health"	43,741	

#6	MH “Help Seeking Behavior”	6,804	
#7	#1 OR #2 OR #3 OR #4 OR #5 OR #6	113,233	
#8	“Mental health literacy” OR “Health literacy” OR literacy OR knowledge OR attitude* OR belief* OR stigma* OR “help-seek*” [AB]	293,937	
#9	#7 OR #8	364,259	Concept 1
CONCEPT 2			
#10	MH “Postnatal Period”	9,240	
#11	MH “Pregnancy”	199,448	
#12	#10 OR #11	203,439	
#13	prenatal OR antenatal OR pregnancy OR “before birth” OR postnatal OR postpartum OR “after birth” OR peripartum OR perinatal [AB]	129,635	
#14	#12 OR # 13	252,475	Concept 2
CONCEPT 3			
#15	MH "Depression"	107,054	
#16	MH "Anxiety”	43,145	
#17	MH “Mental Disorders”	57,678	
#18	MH “Mental Health”	36,848	
#19	#15OR #16 OR #17 OR #18	210,847	
#20	“mental health” OR “mental illness” OR “mental disorder” OR “psychiatric disorder” OR depression OR anxiety OR “baby blues” OR psychosis OR “bipolar disorder” [AB]	226,663	
#21	#19 OR #20	332,673	Concept 3
#22	#9 AND #14 AND #21<br>(Without Filter)	1,822	
#23	#9 AND #14 AND #21<br>(With filter)<br>• English and German	1,768	

4. PSYCINFO SEARCH TERMS

No.	Term	Hits (date: 01.09.2020)	
CONCEPT 1			
#1	DE “Mental Health Literacy”	443	
#2	DE “Health Literacy”	3,508	
#3	DE “Health Knowledge”	7,780	

#4	DE "Health Attitudes"	10,321	
#5	DE "Help Seeking Behavior"	5,659	
#6	#1 OR #2 OR #3 OR #4 OR #5	25,489	
#7	Mental health literacy OR Health literacy OR literacy OR knowledge OR attitude* OR belief* OR stigma* OR "help-seek* [AB]	589,204	
#8	#7 OR #8	597,575	Concept 1
CONCEPT 2			
#9	DE "Postnatal Period"	**4,756**	
#10	DE "Pregnancy"	**45,675**	
#11	DE "Perinatal Period"	**2,907**	
#12	#10 OR #11 OR #12	50,254	
#13	prenatal OR antenatal OR pregnancy OR "before birth" OR postnatal OR postpartum OR "after birth" OR peripartum OR perinatal [AB]	76,167	
#14	#12 OR # 13	91,042	Concept 2
CONCEPT 3			
#15	DE "Major Depression"	124,310	
#16	DE "Anxiety"	80,898	
#17	DE "Mental Disorders"	131,002	
#18	DE "Mental Health"	73,282	
#19	#16 OR #17 OR #18 OR #19	368,894	
#20	"mental health" OR "mental illness" OR "mental disorder" OR "psychiatric disorder" OR depression OR anxiety OR "baby blues" OR psychosis OR "bipolar disorder" [AB]	565,836	
#21	#20 OR #21	673,404	Concept 3
#22	#8 AND #14 AND #21<br>(Without Filter)	1,988	
#23	#8 AND #14 AND #21<br>(With filter)<br>• English and German	1,902	
