# Supplementary material for: Perinatal mental health literacy: knowledge, attitudes, and help-seeking among perinatal women and the public – a systematic review
Source: BMC Pregnancy Childbirth. 2022 Jul 19;22:574. doi: 10.1186/s12884-022-04865-y (PMC9295513; doi:10.1186/s12884-022-04865-y)
Supplement: Supplementary file 3 — Additional file 3. Quality Assessment. This file presents the critical appraisal of the included studies. [file 12884_2022_4865_MOESM3_ESM.pdf]

Perinatal mental health literacy: Knowledge, attitudes, and help-seeking among perinatal women and the public – a systematic review

Critical appraisal of the included cohort and case control studies using the QATSQ tool for non-randomized studies.

Author	Selection bias	Study design	Confounders	Blinding[a]	Data collection	Withdrawals and dropouts	Summary
Logsdon 2018b [76]	[3] Participants are not likely to be representative, response rate was not described	[2] Study is designated as cohort analytic study	[1]Confounders (age, ethnicity, health status, education) were similar across pre and post-test groups	[2]Blinding not described	[1]Tools were shown to be valid and reliable	[1]There was a 96.9-97.9% follow-up rate from those that consented and completed the intervention.	Moderate quality
Small 1994 [79]	[2] Participants are somewhat likely to be representative, response rate was 71.5%	[2] Study is designated as a case control study	[3]Confounders across cases and controls not described	[2]Participants are not aware of the research question	[1]Tools were shown to be valid and reliable	[1]There was a 37.8% follow-up rate from those that consented and scored as depressed after childbirth	Moderate quality
Bina 2014 [68]	[3] Participants are not likely to be representative, response rate was not described	[2] Study is designated as a cohort study	[1]Confounders were similar across follow-up sample and initial sample	-	[1]Tools were shown to be valid and reliable	[1]94% of women screening positive at 6-weeks postpartum participated at a 6-month follow-up	Moderate Quality
O'Mahen 2009 [42]	[3] Participants are not likely to be representative, response rate was 53.9%	[2] Study is designated as a cohort study	[1]Confounders (marital status, age, health status) were similar across follow-up sample and initial sample	-	[1]Tools were shown to be valid and reliable	[1]There was a 73% 6-week postpartum follow-up rate from those that consented	Moderate Quality

The Qualitative Assessment Tool for Quantitative Studies (QATSQ) assesses the domains selection bias, study design, confounding bias, blinding, data collection method and withdrawal/drop-out bias and rates them as weak, moderate or strong; [a]The component ‘blinding of outcome assessors and participants’ was considered not applicable for observational studies in this review; [1] Strong rating; [2] Moderate rating; [3] Weak rating; Strong quality (no weak ratings); Moderate quality (one weak rating); Weak quality (two or more weak ratings); Studies with weak quality were removed from this review.

Critical appraisal of the included RCT studies using the Cochrane Collaboration's Tool

Author	Selection Bias		Performance bias	Detection bias	Attrition bias	Reporting bias	Summary of overall accumulative risk
	Random sequence generation	Allocation concealment	Blinding of participants and personnel	Blinding of outcome assessment	Incomplete outcome data	Selective reporting	
Holt 2017 [74]	[a] Automated computer system used	[a] Allocation schedule produced by an independent researcher	[a] Given the nature of the intervention, Maternal and Child Health Nurses could not be blinded beyond allocation; women were blind to the intervention they received.	[a] The research assistant who collected outcome data was blind	[a] Scores for the observed cases were compared to those of missing cases in terms of the main outcomes at each time point.	[a] All primary outcomes were reported	Low risk of bias
Thorstein sson 2018 [80]	[a] Qualtrics software was used	[b] Unclear who performed randomisation	[b]Unclear if blinding was performed	[b]Unclear whether assessors had knowledge of treatment groups when assessing effects	[a] Missing values were replaced using the replace missing values, series mean method	[a]All outcomes measures were reported, along with effect sizes	Unclear risk of bias

The Cochrane Collaboration's Tool comprises 5 domains of potential bias (selection, performance, detection, attrition and reporting bias). Each domain is judged as high risk of bias, unclear risk of bias or low risk of bias. a Low risk of bias rating; [b]Unclear risk of bias rating; [c] High risk of bias rating; Overall low risk of bias (across all domains low risk is scored); Overall unclear risk of bias (across all domains low or unclear risk of bias is scored); Overall high risk of bias (if one or more domains scored high risk of bias).

Critical appraisal of the included cross-sectional studies using the NOS tool.

Authors	Selection				Outcome		Summary
	**Representativeness of the sample (sampling method)**<br>a. Truly representative of the average in the target population. * (all subjects or random sampling)<br>b. Somewhat representative of the average in the target group. * (non-random sampling)<br>c. Selected group of users/convenience sample.<br>d. No description of the derivation of the included subjects.	**Sample size**<br>a. Justified and satisfactory (including sample size calculation). *<br>b. Not justified.<br>c. No information provided	**Response rate (%)**<br>a. The minimum satisfactory response rate was defined as 60% for paper-based surveys and 30% for web based surveys*<br>b. Unsatisfactory recruitment rate, no summary data on non-respondents.<br>c. No information provided	**Ascertainment of the exposure (risk factors: history of mental illness /current mental illness)**<br>a) Validated measurement tool. **,<br>b) Non-validated measurement tool, but the tool is available or described *,<br>c) No description of the measurement tool / not measured.	**Assessment of the outcome**<br>a)Independent blind assessment. **,<br>b) Record linkage. **<br>c) Self report. *<br>and d) No description	**Statistical test**<br>a. Statistical test used to analyse the data clearly described, appropriate and measures of association presented including confidence intervals and probability level (p value). *<br>b. Statistical test not appropriate, not described or incomplete.	
Ayres 2019 [67]	c	Not justified	a* (71%)	b*	Self-report*	described*	4
Azale 2016 [40]	b*	Not justified	a* (100%)	a**	Self-report*	described*	6
Barrera 2015 [33]	c	Not justified	b (26.38%)	a**	Self-report*	described*	4
Branquinho 2019 [26][a]	c	Not justified	c	b*	Self-report*	described*	3
Branquinho 2020* [53][a]	c	Not justified	c	b*	Self-report*	Described*	3
Buist 2005[30]	c	Not justified	b (46%)	a**	Self-report*	Described*	5

Buist 2007 [20]	c	Not justified	b (57%)	a**	Self-report*	Described*	4
DaCosta 2018 [38]	c	Not justified	a* (55.4%; internet survey)	a**	Self-report*	Described*	4
Dunford 2017 [37]	c	Not justified	a* (77%; internet survey)	a**	Self-report*	Described*	5
Fonseca 2017 [69][a]	c	Not justified	c	a**	Self-report*	Described*	4
Fonseca 2015 [39][a]	c	Not justified	c	a**	Self-report*	Described*	4
Fonseca 2018 [70][a]	c	Not justified	c	a**	Self-report*	Described*	4
Ford 2019 [71]	c	Not justified	c	a**	Self-report*	Described*	4
Goodman 2009[34]	c	Not justified	c	a**	Self-report*	Described*	4
Goodman 2013 [72]	c	Not justified	c	a**	Self-report*	Described*	4
Henshaw 2013 [73]	c	Not justified	c	a**	Self-report*	Described*	4
Highet 2011 [18]	a*	Not justified	13%	c	Self-report*	Described*	3
Kim 2010 [41]	c	Justified but no sample size calculation	c	a**	Self-repot*	Described*	4
Kingston 2014a [75][a]	a*	Not justified	27.6%	c	Self-report*	Described*	3
Kingston 2014b [17][a]	a*	Not justified	27.6%	c	Self-report*	Described*	3
Logsdon 2018a [43]	c	Not justified	c	a**	Self-report*	Described*	4

Mirsalimi 2020 [47]	c	Justified and calculated*	c	c	Self-report*	Described*	3
O'Mahen 2008 [77]	c	Not justified	62%*	a**	Self-report*	Described*	5
Patel 2011 [78]	c	Not justified	39%*	b*	Self-report*	Described*	4
Prevatt 2018 [59]	c	Not justified	73%*	a**	Self-report*	Descried*	5
Ride 2016 [31][b]	c	justified	c	c	Self-report*	Described*	3
Sealy 2009 [19]	b*	Not justified	57-62%*	c	Self-report*	Described*	4
Sleath 2005 [35]	c	Not justified	91%*	a**	Self-report*	Described*	5
Smith 2019 [28]	b*	Not justified	c	c	Self-report*	Described*	3
Thorsteinsson 2014 [29]	c	Not justified	c	a*	Self-report*	Described*	3
Wenze 2018 [36]	c	Not justified	c	a**	Self-report*	Described*	4
Zittel-Palamara 2008 [32]	c	Not justified	c	b*	Self-report*	Described*	3

The Newcastle-Ottawa Scale (NOS) assesses three domains of bias: selection, comparability and outcome biases. As all included cross-sectional studies were descriptive studies, the comparability section was omitted in this review, resulting in 2 domains with a maximum of 8 points. Studies below 2 points were considered to indicate poor quality, 3-6 points considered to indicate fair quality and more than seven points are considered to indicate good quality; * one score obtained; ** two score obtained; Good quality (score of ≥ 7); Fair quality (score 3 to 6); Poor quality (score of ≤ 2); Studies with poor quality were removed from this review; [a]same sample used in more than one paper; [b]cross-sectional internet survey including a discrete choice experiment.
