# Supplementary material for: Perinatal mental health literacy: knowledge, attitudes, and help-seeking among perinatal women and the public – a systematic review
Source: BMC Pregnancy Childbirth. 2022 Jul 19;22:574. doi: 10.1186/s12884-022-04865-y (PMC9295513; doi:10.1186/s12884-022-04865-y)
Supplement: Supplementary file 4 — Additional file 4. Supplemental Results. This file presents the results relating to overall levels of perinatal mental health literacy. [file 12884_2022_4865_MOESM4_ESM.pdf]

Perinatal mental health literacy: Knowledge, attitudes, and help-seeking among perinatal women and the public – a systematic review

Supplemental Results

Overall levels of perinatal MHL

Only two studies reported on levels of MHL in relation to PMHP. Both studies focused on postpartum depression literacy with one study assessing postpartum depression literacy among the public [53] and one study in perinatal women [47]. The study assessing postpartum depression literacy in perinatal women classified their participants as having moderate levels of postpartum depression literacy.

Tabel S2 Levels of perinatal mental health literacy

**Authors**	**Perinatal mental health literacy levels**
Branquinho et al., 2020[a] [53]	Postpartum depression literacy: M=10.45; SD=2.74
Mirsalimi, Ghofranipour, Noroozi & Montazeri, 2020[c] [47]	Postpartum depression literacy: M=3.79; SD=0.39; moderate level of depression literacy

[a]The Knowledge about Postpartum Depression Questionnaire (KPPD-Q) (score range 0-15; higher scores indicate higher postpartum depression knowledge)
[c] Postpartum Depression Literacy Scale (PoDLiS); 31 items (range 1-5)
