# Supplementary material for: Perinatal mental health literacy: knowledge, attitudes, and help-seeking among perinatal women and the public – a systematic review
Source: BMC Pregnancy Childbirth. 2022 Jul 19;22:574. doi: 10.1186/s12884-022-04865-y (PMC9295513; doi:10.1186/s12884-022-04865-y)
Supplement: Supplementary file 5 — Additional file 5. Tools to measure perinatal mental health literacy components. This file summarizes the tools used in the included studies [86–90]. [file 12884_2022_4865_MOESM5_ESM.pdf]

Perinatal mental health literacy: Knowledge, attitudes, and help-seeking among perinatal women and the public – a systematic review

Table S1 Measures of the perinatal MHL components

Measures	Developer	Studies using the tool	Number of studies using the tool (%)
**Knowledge Measures**			**13 (100)***
Vignette-based questions		Buist 2005 [30](a,g); Buist 2007 [20] (a), Thorsteinsson 2014 [29](a)	3 (23.1)
Short Explanatory Model Interview (SEMI)	Lioyd 1988 [86]	Azale 2016 [40](c, g)	1 (7.7)
Knowledge about Postpartum Depression Questionnaire (KPPD-Q)	Branquinho 2019 [26]	Branquinho 2019 [26](b, c, g)	1 (7.7)
Single (Open end) questions		Henshaw 2013 [73](c), Branquinho 2019 [26](a)	2 (15.4)
Perinatal Depression Monitor	Highet 2011 [18]	Highet 2011 [18](a,b,c), Kingston 2014b [17](b, c), Smith 2019 [28](a, b,c,g)	3 (23.1)
Attitudes Toward Seeking Professional Psychological Help Scale (ATSPPHS)	Fischer 1995 [87]	Logsdon 2018a [43](f)	1 (7.7)
The Illness Perception Questionnaire; IPQ)	Weinman 1996 [88]	O'Mahen 2009 [42](c)	1 (7.7)
Study specific measures (questionnaire / interview)		Sealy 2009 [19](b,g), Small 1994 [79](c), Thorsteinsson 2014 [29](c,g)	3 (23.1)
**Attitudes (Stigma / Belief Measures)**			**7 (100)***
List of statements		Smith 2019 [28](l)	1 (14.3)
Attitudes about Postpartum Depression Questionnaire (APPD-Q)	Branquinho 2019 [26]	Branquinho 2019 [26](l), Branquinho 2020 [53](l)	2 (28.6)
Stigma subscale of the Portuguese version of the Inventory of Attitudes Toward Seeking Mental Health Services (IATSMHS)	Fonseca 2017 [27]	Branquinho 2020 [53](l), Fonseca 2018 [70](h,l)	2 (28.6)
The Inventory of Attitudes Towards Seeking Mental Health Services (IASMHS); stigma subscale (indifference to stigma)	Mackenzie 2004 [82]	Dunford 2017 [37](l)	1 (14.3)
Perinatal Depression Monitor	Highet 2011 [18]	Highet 2011 [18](l)	1 (14.3)
Depression Stigma Scale (DSS)	Griffiths 2004 [55]	Thorsteinsson 2018 [80](l)	1 (14.3)
**Help-seeking Measures**			**34 (100)***
Single (open-end) question		Ayres 2019 [67](h), Barrera 2015 [33](i), Buist 2005 [30] (i); DaCosta 2018 [38](i), Fonseca 2018 [70](h), Goodman 2013 [72](h; k, facilitators), Logsdon 2018a [43](j), Logsdon 2018b [76] (h), Ride 2016 [31](h), Smith 2019 [28](h, i)	10 (29.4)
General Help Seeking Questionnaire (GHSQ)	Wilson 2005 [83]	Azale 2016 [40](h, i), Fonseca 2017 [69] (h), Fonseca 2018 [70] (h)	3 (8.8)
Barriers to Access to Care Evaluation (BACE)	Clement 2012 [89]	Azale 2016 [40](k)	1 (2.9)
List of statements (e.g., barriers, treatment options)		Ayres 2019 [67](k), Barrera 2015 [33](k), Bina 2014 [68] (i), DaCosta 2018 [38](k), Fonseca 2015 [39](k; based on the Barriers Scale; O'Mahen 2008 [77]), Goodman 2009 [34](j,k), Goodman 2013 [72](j; k facilitators), Holt 2017 [74] (i,k), Kingston 2014a [75](j), O'Mahen 2008 [77](i,j,k), Patel 2011 [78](j), Prevatt 2018 [59](i), Sleath 2005 [35](j), Zittel-Palamara 2008 [32](i,j,k)	14 (41.2)

Attitudes Toward Seeking Professional Psychological Help Scale (ATSPPHS)	Fischer 1995 [87]	Bina 2014 [68](k)	1 (2.9)
Inventory of Attitudes Towards Seeking Mental health Services; help-seeking propensity (IASMHS)	Mackenzie 2004 [82]	Dunford 2017 [37](h), Thorsteinsson 2018 [80](h)	2 (5.9)
The Help-seeking Propensity subscale of the Portuguese version of the Inventory of Attitudes Toward Seeking Mental Health Services (IATSMHS)	Fonseca 2017 [27]	Branquinho 2020 [53](h)	1 (2.9)
Study specific measure (questionnaire / interview)		Ford 2019 [71](k), Goodman 2009[34] (i), Goodman 2013 [72] (k), Henshaw 2013 [73] (i), Mirsalimi 2020 [47] (i),Thorsteinsson 2014 [29] (i,j), Wenze 2018 [36](h, j, k), Kim 2010 [41](k), Small 1994 [79] (h,i)	9 (26.5)
Perinatal Depression Monitor	Highet 2011 [18]	Highet 2011 [18] (i), Kingston 2014a [75](i)	2 (5.9)
Stigma Scale for Receiving Psychological Help (SSRPH)	Komiya 2000 [90]	Logsdon 2018a [43](k)	1 (2.9)
Mental Health Intention Scale (1 item)	Gerend 2006 [84]	Logsdon 2018a [43](i)	1 (2.9)
The Perceived Barriers to Psychological Treatment (PBPT)	Mohr 2010 [46]	Prevatt 2018 [59] (k)	1 (2.9)
Discrete Choice experiment (mixed logit model)		Ride 2016 [31](i, k)	1 (2.9)
logistic regression / path model to examine which factors predict help-seeking or diclosure		Azale 2016 [40] (k), Barrera 2015 [33] (k), Bina 2014 [68] (k), DaCosta 2018 [38](k), Fonseca 2018 [70](k), Holt 2017 [74] (k), O'Mahen 2009 [42] (k), Prevatt 2018 [59] (k, facilitators)	8 (23.5)
**MHL levels regarding PMHP**			2 **(100)***
Knowledge about Postpartum Depression Questionnaire (KPPD-Q)	Based on the Depression Literacy Questionnaire (Griffiths 2004 [55])	Branquinho 2020 [53]	1 (50)
Postpartum depression Literacy Scale (PoDLiS)	Mirsalimi 2020 [47]	Mirsalimi 2020 [47]	1 (50)

N/A, not available; a, Recognition of disorder; b, Symptoms; c, Causes; e, First aid/self -help; f, Prevention; g, Intervention; h, Help-seeking intention; i, preferred source of help; j, preferred treatment; k, barriers and/or facilitators to help -seeking; l, Stigmatizing attitudes and beliefs towards perinatal mental illness; * As some studies used multiple tools in one category (e.g., knowledge measures), the sum of all percentages could add up to more than 100%
